# Integrative Genomics and Computational Systems Medicine

**DOI:** 10.1155/2014/945253

**Published:** 2014-06-15

**Authors:** Jason E. McDermott, Yufei Huang, Bing Zhang, Hua Xu, Zhongming Zhao

**Affiliations:** ^1^Computational Biology and Bioinformatics Group, Pacific Northwest National Laboratory, Richland, WA 99352, USA; ^2^Department of Electrical and Computer Engineering, The University of Texas at San Antonio, San Antonio, TX 78249, USA; ^3^Department of Biomedical Informatics, Vanderbilt University School of Medicine, Nashville, TN 37203, USA; ^4^Center for Quantitative Sciences, Vanderbilt University Medical Center, Nashville, TN 37232, USA; ^5^Department of Cancer Biology, Vanderbilt University School of Medicine, Nashville, TN 37232, USA; ^6^School of Biomedical Informatics, The University of Texas Health Science Center at Houston, Houston, TX 77030, USA

The exponential growth in generation of large amounts of genomic data from biological samples has driven the emerging field of systems medicine. This field is promising because it improves our understanding of disease processes at the systems level. However, the field is still in its young stage. There exists a great need for novel computational methods and approaches to effectively utilize and integrate various -omics data.

Systems medicine has been growing rapidly in part due to the emerging technologies to gather high-volume measurements from biological samples. One of the first such technologies, the mRNA microarray, is being replaced by next generation sequencing (NGS), which provides a much higher resolution (digital measurement) of genetic information (e.g., at the mRNA transcript level). Array and NGS-based methods to characterize genetic variation (single nucleotide polymorphisms, short insertions and deletions, copy number variation, and structural variants), DNA methylation changes, microRNAs (miRNAs) differential expression, and other types of biological information have dramatically expanded the generation of biological data. Other sources of data from mass spectrometry-based proteomics and metabolomics to high-throughput determination of protein-protein interactions and regulatory relationships provide further information for a systems-level understanding of disease. Finally, collection of clinical data and electronic medical records (EMRs) has made modern biomedical research possible on the full scale of data integration, that is, an integration scenario of using genomic, transcriptomic, proteomic, metabolomic, and phenotypic data.

The rationale of discovery-based high-throughput investigation of disease is that there are molecular signatures (composed of genes, transcripts, proteins, and small molecules) that can be identified for better diagnosis, prognosis, and/or treatment of disease. However, challenges arise in the analysis of high-throughput data because of the large number of possible variables raising the very real potential for false-positive predictions and overfitting of data, as well as many other potential problems (e.g., data quality, missingness, lack of power, etc.). To ameliorate these problems, computational approaches have been developed that utilize existing knowledge, such as overlaying high-throughput observations on regulatory or protein-protein interaction networks or canonical biological pathways.

For this special issue we solicited manuscripts in several different subject areas including data integration from multiple high-throughput sources, NGS data analysis and applications, personalized medicine and translational bioinformatics, modeling of pathways and networks, and data mining and pattern recognition in biomedical applications. We briefly describe the accepted papers in this special issue in the remainder of this editorial.

Two papers, “*MultiRankSeq: multi-perspective approach for RNAseq differential expression analysis and quality control*” by Y. Guo et al. and “*QPLOT: a quality assessment tool for next generation sequencing data*” by B. Li et al., describe algorithms for analysis of NGS data. Y. Guo et al. introduce a novel tool, namely, MultiRankSeq, which combines the output of three independent programs to determine differential expression from RNAseq data to provide a single improved output. QPLOT is a tool for assessing the quality of NGS runs by providing both summary quality metrics and graphical representations of these metrics. In another paper, entitled “*Comparative study of exome copy number variation estimation tools using array comparative genomic hybridization as control*,” Y. Guo et al. systematically compare four different tools for detecting copy number variations (CNVs) from whole exome sequencing (WES) against a standard array-based method for CNV evaluation.

In “*Computational analysis of transcriptional circuitries in human embryonic stem cells reveals multiple and independent networks*,” X. Wang and C. Guda assess the role of core transcription factors in the pluripotency of embryonic stem (ES) cells. Their computational analyses identified several additional transcriptional regulatory networks that might be involved in this complex regulatory process, providing interesting hypotheses about mechanisms of fate determination in ES cells. The paper “*Network-assisted prediction of potential drugs for addiction*” by J. Sun et al. describes computational analyses of drug-target networks for addictive and nonaddictive drugs. The authors analyzed the topology of these networks and found that drugs with similar effects could cluster together and identified a set of nonaddictive drugs that might have therapeutic benefits for treatment of addiction. This paper was called out in the recent “Translational Bioinformatics Year-in-Review” in 2014 Joint Summits on Translational Science (http://www.amia.org/jointsummits2014). In “*DeGNServer: deciphering genome-scale gene networks through high performance reverse engineering analysis*,” J. Li et al. describe their webserver to infer transcriptional regulatory networks from large-scale datasets. The server makes use of a computer cluster to run a number of network inference algorithms and return the results to the user very quickly, thus facilitating genome-scale network reconstruction.

Two papers from M. Goyal et al., “*Development of dual inhibitors against Alzheimer's disease using fragment-based QSAR and molecular docking*” and “*Novel natural structure corrector of ApoE4 for checking Alzheimer's disease: benefits from high throughput screening and molecular dynamics simulations*,” deal with molecular docking simulations to determine small-molecule inhibitors targeting Alzheimer's disease. In the first paper, M. Goyal et al. used a fragment-based quantitative structure activity relationship (QSAR) analysis to identify lead compounds that might inhibit interaction of proteins that drive Alzheimer's disease pathogenesis. In the second paper, the authors describe large-scale docking simulations to screen for inhibitors of the conformational change of apolipoprotein E4 (ApoE4) that is thought to drive Alzheimer's pathogenesis. They further show the value of molecular dynamics simulations to screen candidates to eliminate molecules that do not have stable binding properties with targets. In “*HGF accelerates wound healing by promoting the dedifferentiation of epidermal cells through β*_1_ *-integrin/ILK pathway*,” J.-F. Li et al. experimentally investigate the contribution of hepatocyte growth factor (HGF) to wound healing. They showed that treatment of diabetic mice promoted proliferation and migration of epithelial cells and that this effect could be blocked by silencing the *β*_1_-integrin signaling pathway.

In the paper “*Integrative analysis of miRNA-mRNA and miRNA-miRNA interactions*,” the authors first generated RNAseq data for normal and tumor cell lines and then identified aberrantly expressed mRNAs and miRNAs. Groups of similarly expressed miRNAs and mRNAs were analyzed to highlight examples of flexible and selective regulatory networks underlying these interactions. In “*A diverse stochastic search algorithm for combination therapeutics*,” M. U. Caglar and R. Pal show how the use of a stochastic search algorithm can be useful in identification of optimal combinations of drugs for therapy, the so-called drug cocktails. Their novel method greatly reduces the number of experimental steps needed to assess the optimal combination of drugs for a particular therapy. In “*Evaluating word representation features in biomedical named entity recognition tasks*” by B. Tang et al., the authors present a comparative analysis of three different methods for word representation in recognition of named entities from biomedical literature. Their findings indicate that a combination of the complementary approaches can improve results on benchmark recognition tasks.

In the paper by F. Zhang et al., “*Multiple biomarker panels for early detection of breast cancer in peripheral blood*,” the authors describe the use of machine-learning approaches to identify a five-gene panel that can identify breast cancer from peripheral blood samples. In the paper by Jiang et al., “*New aQTL SNPs for the CYP2D6 identified by a novel mediation analysis of genome-wide SNP arrays, gene expression arrays, and CYP2D6 activity*,” the authors develop a novel approach for the detection of transexpression quantitative trait loci (eQTLs) from genome-wide association studies by considering indirect effects introduced by a mediator gene. They apply their method to analyze indirect regulatory effects on the important liver enzyme, CYP2D6. Finally, in “*Expression sensitivity analysis of human disease related genes*,” L.-X. Ma et al. examine the expression of genes implicated in a range of diseases. They report that genes that are robustly expressed under different perturbations are more likely to be associated with lethal diseases, whereas less robustly expressed genes are associated with nonlethal diseases.

